# The Multifaceted Actions of PVP–Curcumin for Treating Infections

**DOI:** 10.3390/ijms25116140

**Published:** 2024-06-02

**Authors:** Magdalena Metzger, Stefan Manhartseder, Leonie Krausgruber, Lea Scholze, David Fuchs, Carina Wagner, Michaela Stainer, Johannes Grillari, Andreas Kubin, Lionel Wightman, Peter Dungel

**Affiliations:** 1 Ludwig Boltzmann Institute for Traumatology, The Research Center in Cooperation with AUVA, 1200 Vienna, Austria; 2 Austrian Cluster for Tissue Regeneration, 1200 Vienna, Austria; 3 Institute of Molecular Biotechnology, University of Natural Resources and Life Sciences, 1190 Vienna, Austria; 4 Planta Naturstoffe Vertriebs GmbH, 1230 Vienna, Austria

**Keywords:** antimicrobial resistance, antimicrobial photodynamic therapy, bacterial SOS response

## Abstract

Curcumin is a natural compound that is considered safe and may have potential health benefits; however, its poor stability and water insolubility limit its therapeutic applications. Different strategies aim to increase its water solubility. Here, we tested the compound PVP–curcumin as a photosensitizer for antimicrobial photodynamic therapy (aPDT) as well as its potential to act as an adjuvant in antibiotic drug therapy. Gram-negative *E. coli* K12 and Gram-positive *S. capitis* were subjected to aPDT using various PVP–curcumin concentrations (1–200 µg/mL) and 475 nm blue light (7.5–45 J/cm^2^). Additionally, results were compared to aPDT using 415 nm blue light. Gene expression of *recA* and *umuC* were analyzed via RT-qPCR to assess effects on the bacterial SOS response. Further, the potentiation of Ciprofloxacin by PVP–curcumin was investigated, as well as its potential to prevent the emergence of antibiotic resistance. Both bacterial strains were efficiently reduced when irradiated with 415 nm blue light (2.2 J/cm^2^) and 10 µg/mL curcumin. Using 475 nm blue light, bacterial reduction followed a biphasic effect with higher efficacy in *S. capitis* compared to *E. coli* K12. PVP–curcumin decreased *recA* expression but had limited effect regarding enhancing antibiotic treatment or impeding resistance development. PVP–curcumin demonstrated effectiveness as a photosensitizer against both Gram-positive and Gram-negative bacteria but did not modulate the bacterial SOS response.

## 1. Introduction

Antimicrobial resistance poses a significant threat to global health, rendering diseases caused by various microorganisms increasingly difficult to treat. This includes skin and soft tissue infections, leading to severe complications if not treated effectively. The persistent presence of microbes can cause a permanent and prolonged inflammatory immune response, which may delay recovery and even cause tissue damage [1].

The conventional treatment of bacterial infections relies on the use of antibiotic drugs; however, the rise of resistant strains necessitates exploring alternative treatment options. Antimicrobial photodynamic therapy (aPDT) emerges as a promising approach, employing light and a photosensitizer (PS) to generate reactive oxygen species (ROS) that inactivate microbial cells. Notably, due to its unspecific mode of action, aPDT also exhibits efficacy against multi-drug-resistant bacterial strains and has been effective against Gram-negative as well as Gram-positive strains [2,3,4].

Curcumin is a polyphenol isolated from the rhizome of *Curcuma longa*. It has been reported to exhibit photosensitizing capabilities; thus, it is potentially suitable for aPDT [3,5]. However, the molecule is rather unstable and has a poor water-solubility which limits its therapeutic applications. Recently, strategies to overcome these problems have been undertaken, including the complexation with polyvinylpyrrolidone (PVP).

Besides the inherent properties of the PS, for aPDT the appropriate light source is of equal importance. Curcumin absorbs in the blue light range with a peak in absorbance at 416 nm. Therefore, the closer the wavelength used is to this maximum, the better the effectiveness. However, there is evidence that particularly longer wavelengths in the blue light range possess wound-healing-promoting properties, mostly due to pro-angiogenic and anti-inflammatory effects [6,7,8,9]. Thus, the combination of the parameters of light treatment could be taken into consideration.

In addition, besides acting as a PS, curcumin has also been reported to potentially affect the bacterial SOS response, a DNA repair mechanism activated by DNA damage [10,11,12]. The protein RecA, a key player in this pathway, recognizes single-stranded DNA regions, binds them, and forms long nucleofilaments to protect the DNA while also binding to the transcriptional repressor LexA. This induces the auto-proteolysis of LexA and facilitates the expression of the so-called SOS genes, most of which are needed for DNA repair, homologous recombination, cell cycle arrest and replication [13,14,15]. They also include the gene encoding for RecA (*recA*), but also proteins that can contribute to the development of antibiotic resistances such as the error-prone DNA polymerase V (subunits encoded by *umuC* and *umuD*) [16]. Therefore, inhibiting the protein RecA has been recently proposed as a promising strategy to deprive bacterial cells of DNA repair mechanisms and prevent the emergence of drug resistances.

Building on these insights, the aim of this work was to investigate the multifaceted actions of the water-soluble curcumin variant PVP–curcumin, both as a photosensitizer as well as a RecA inhibitor. Combining the antibacterial mechanism of aPDT while, at the same time, depleting bacteria of repair mechanisms and survival strategies could significantly improve the treatment of infected wounds.

## 2. Results

### 2.1. PVP–Curcumin Acted as a Photosensitizer when Irradiated with Blue Light in a Concentration- and Light-Dose-Dependent Manner

**Figure 1:**
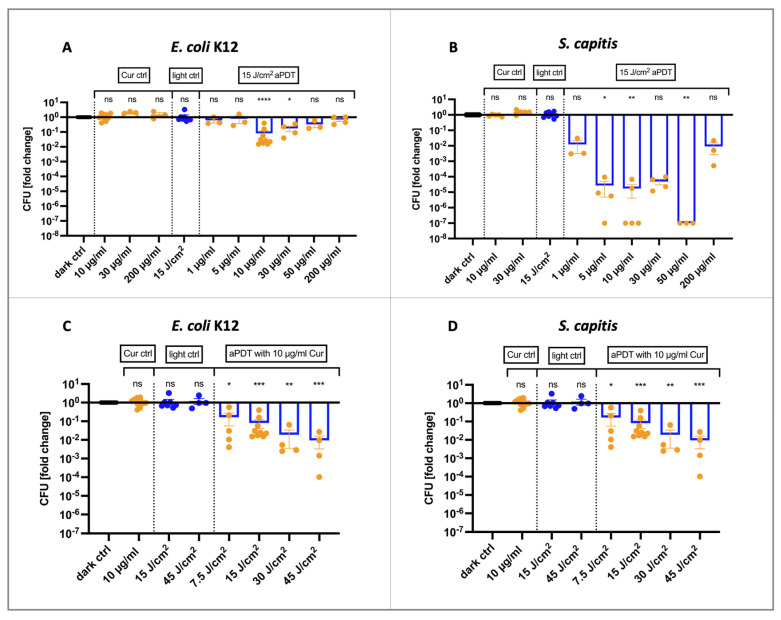
Antibacterial effects of photodynamic therapy using blue light (475 nm). (**A**) When the Gram-negative bacterial strain *E. coli* K12 was irradiated with 15 J/cm^2^, it was most efficiently decreased with 10 µg/mL curcumin (LRV = 1.09, 91.83%, *p* < 0.0001). Higher concentrations were less effective. Curcumin concentrations of up to 200 µg/mL did not affect bacterial growth without photoactivation. (**B**) Colony-forming units (CFUs) of *S. capitis* were already decreased by 1.9-log_10_ units (98.76%, *p* = 0.81) when irradiated with 15 J/cm^2^ after incubation with 1 µg/mL curcumin. Compared to *E. coli* K12, aPDT efficacy using 10 µg/mL curcumin was higher in *S. capitis* with LRV = 4.76 (99.998%, *p* = 0.0038). Antibacterial effects of aPDT decreased with 200 µg/mL curcumin (LRV = 2.04, 99.1%, *p* = 0.7544). Increasing the light dose when treating bacterial cells with 10 µg/mL curcumin enhanced therapy outcome (**C**,**D**). A total of 15 and 45 J/cm^2^ of 475 nm blue light alone did not have an effect on the growth of neither *E. coli* K12 nor *S. capitis.* Mean ± SEM, *n* = 3–10, Kruskal–Wallis test with Dunn’s multiple comparisons test, * *p* ≤ 0.05, ** *p* ≤ 0.01, *** *p* ≤ 0.001, **** *p* ≤ 0.0001.

Treating a Gram-negative (*Escherichia coli* K12) and a Gram-positive (*Staphylococcus capitis*) bacterial strain with increasing concentrations of PVP–curcumin and irradiating with a fluence of 15 J/cm^2^ blue light (475 nm) yielded a reduction of colony-forming units (CFUs) following a biphasic dose response curve (Figure 1A,B). Interestingly, aPDT, with the lowest as well as with the highest concentrations of PVP–curcumin, caused the lowest decrease in bacterial numbers. In the case of *E.coli* K12, the strongest inactivation was achieved with 10 µg/mL curcumin (mean log_10_ reduction values (LRV) = 1.09, 91.83%, *p* < 0.0001). Increasing the light dose from 15 to 45 J/cm^2^ led to a CFU reduction of 2.02-log_10_ units (99.05%, *p* = 0.0009, Figure 1C). Notably, the photoinactivation was more effective in Gram-positive *S. capitis* (Figure 1B). Here, 5 µg/mL curcumin caused a mean decrease of 4.56-log_10_ units (99.997%, *p* = 0.0185), while LRV_50 µg/mL_ = 7.0 (99.99999%, *p* = 0.0096). In contrast, the reduction using 200 µg/mL was only 2.04-log_10_ units (99.1%, *p* = 0.7544) on average. Photoinactivation could also be enhanced by increasing the light fluence, as shown with 10 µg/mL curcumin in Figure 1D. The effects of 475 nm blue light alone as well as all tested concentrations of curcumin alone did not differ from the untreated control group. Hence, curcumin could act as an efficient PS regarding Gram-positive bacterial strains. On the other hand, these data suggest that a sufficient reduction of Gram-negative strains requires higher light doses.

### 2.2. aPDT Using 415 nm Blue Light Showed Strong Antibacterial Effects in E. coli K12 and S. capitis

**Figure 2:**
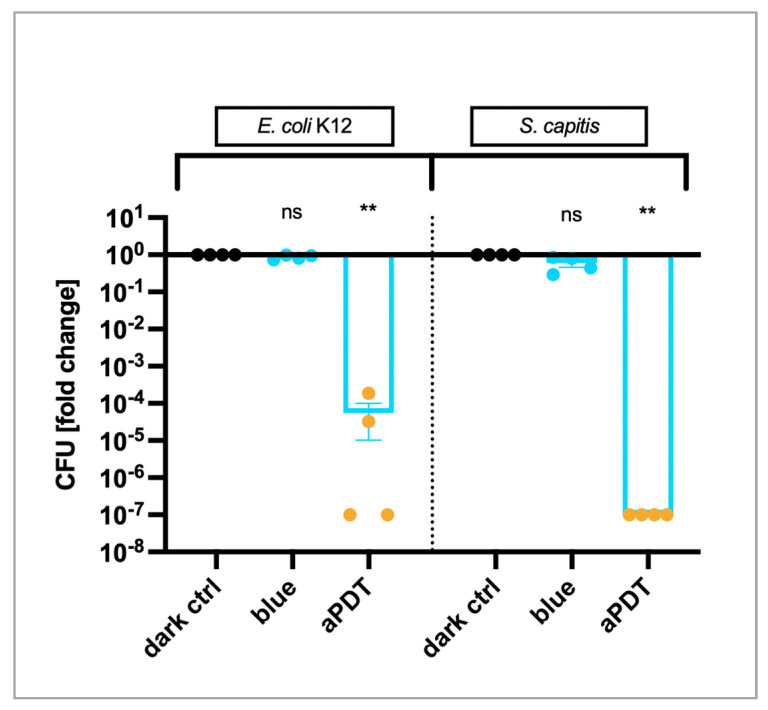
aPDT with 10 µg/mL curcumin and 2.2 J/cm^2^ of 415 nm blue light. CFUs of *E. coli* K12 were significantly reduced by aPDT (LRV = 4.26, 99.994%, *p* = 0.0027) but not by 415 nm blue light alone. Similarly, a decrease of 7.0-log_10_ units (99.99999%, *p* = 0.0023) was observed in *S. capitis*. This Gram-positive bacterial strain was also not affected by 415 nm blue light alone. Mean ± SEM, *n* = 4, Kruskal–Wallis test with Dunn’s multiple comparisons test, ** *p* ≤ 0.01.

As 415 nm blue light is almost at the reported absorbance maxima (416 and 432 nm, Supplemental Figure S1) in contrast to 475 nm, the experiments were repeated with this shorter wavelength. The aPDT of both bacterial strains (*E. coli* K12 and *S. capitis*) using 10 µg/m curcumin and 2.2 J/cm^2^ of 415 nm blue light led to a significant decline in CFUs. *E. coli* K12 was decreased by 4.26-log_10_ units (99.994%, *p* = 0.0027) while the LRV of *S. capitis* was 7.0 (99.99999%, *p* = 0.0023). Blue light alone with a wavelength of 415 nm did not show any adverse effects on bacterial growth (see Figure 2). Hence, aPDT using 415 nm was more effective than 475 nm, which was especially noticeable in the Gram-negative strain.

### 2.3. PVP–Curcumin Decreased Expression of recA in E. coli K12

**Figure 3:**
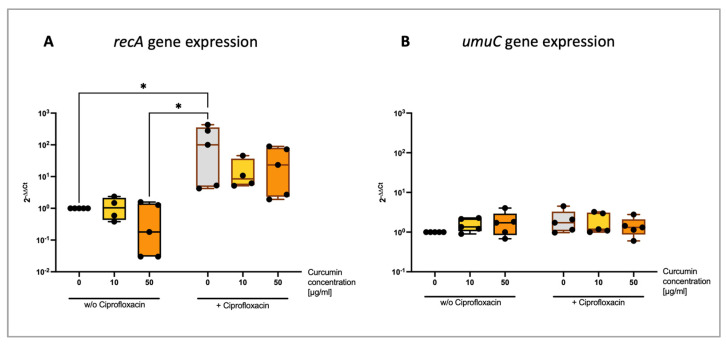
Gene expression of *recA* and *umuC* after pre-incubation of *E. coli* K12 with PVP–curcumin and subsequent Ciprofloxacin treatment. (**A**) PVP–curcumin had little effect on the expression of the gene *recA* in non-stressed cells with a slight decrease in expression when exposed to 50 µg/mL curcumin (2^−ΔΔCt^ = 0.62, *p* > 0.9999). When a sublethal dose of Ciprofloxacin was added, the expression increased on average 163.73-fold (*p* = 0.049). This effect was less pronounced when cells were first pre-treated with 10 µg/mL curcumin (2^−ΔΔCt^ = 16.91, *p* = 0.3465) and 50 µg/mL curcumin (2^−ΔΔCt^ = 37.93, *p* = 0.2803). (**B**) There was no increase in *umuC* expression detected among all experimental groups. Housekeeping gene: *yccT*. Box and whisker blots indicating median, minimum and maximum values, Kruskal–Wallis test, Dunn’s multiple comparison test, ROUT method outlier test, *n* = 5, * *p* ≤ 0.05.

There are reports that curcumin not only has PS properties but can also directly affect bacteria by inhibiting the internal SOS response [10,11,12]. The expression of SOS response-relevant genes *recA* and *umuC* were determined after sublethal Ciprofloxacin treatment with or without 20 min pre-incubation with PVP–curcumin (0, 10 or 50 µg/mL curcumin concentration) (Figure 3). The Ciprofloxacin-induced expression of the gene *recA* (mean 2^−ΔΔCt^ = 163.73, *p* = 0.049) tended to be lower when bacterial cells were first treated with 10 µg/mL curcumin (2^−ΔΔCt^ = 16.91, *p* = 0.3465) or 50 µg/mL curcumin (2^−ΔΔCt^ = 37.93, *p* = 0.2803). Additionally, a slight but not significant trend towards decreased *recA* expression was also observed in the 50 µg/mL group that was not treated with Ciprofloxacin (2^−ΔΔCt^ = 0.62, *p* > 0.9999). It is noteworthy to mention that the average elevation of Ciprofloxacin-induced *recA* expression was 163.73-fold in relation to the control group, however, there was a strong variation between experimental runs ranging from a 4.23-fold to 430-fold increase of the *recA* expression. Still, the trend towards a lower *recA* expression when the cells were pre-treated with curcumin was noticeable in all experimental runs. This effect is more perceptible when comparing the *recA* expression levels of the combination treatments to the Ciprofloxacin-only group instead of the untreated control group. There, the average decrease was 0.73-fold with 10 µg/mL curcumin and 0.41-fold with 50 µg/mL (see Supplemental Material Figure S3). No difference of *umuC* expression was detected among all tested parameters.

### 2.4. PVP–Curcumin Increased Antibacterial Efficacy of Ciprofloxacin in E. coli K12 Depending on Concentration and Incubation Time

**Figure 4:**
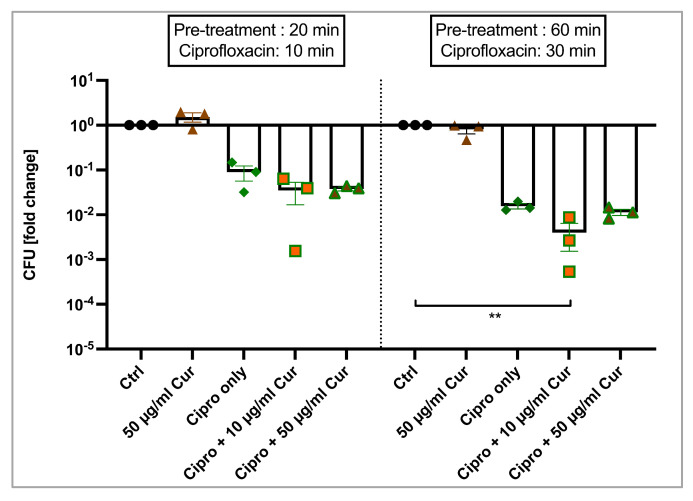
Potentiation of sublethal Ciprofloxacin treatment in *E. coli* K12. A minor increase from 1.81- to 2.4-log_10_ units of antibacterial efficacy was observed when cells were pre-treated with 10 µg/mL curcumin for 60 min. Ctrl indicates an untreated control group, Cur stands for curcumin and Cipro only stands for Ciprofloxacin treatment. Kruskal–Wallis test, Dunn’s multiple comparison test, *n* = 3, ** *p* ≤ 0.01.

*E. coli* K12 was pre-treated with 10 or 50 µg/mL curcumin for either 20 or 60 min before being exposed to a sublethal concentration of Ciprofloxacin (for 10 or 30 additional minutes, respectively). Visualized in Figure 4, Ciprofloxacin alone reduced the bacterial count on average by 1.05-log_10_ units (91.03%, *p* = 0.5708) in the shorter incubation protocol (10 min Ciprofloxacin). LRV after pre-treatment (20 min) with 10 µg/mL or 50 µg/mL curcumin was similar (LRV_10 µg/mL_ = 1.46, 96.52%, *p* = 0.1116; LRV_50 µg/mL_ = 1.42, 96.21%, *p* = 0.1404). A slight decrease of CFUs was observed in the longer treatment protocol with 10 µg/mL curcumin + Ciprofloxacin (LRV = 2.4, 99.6%, *p* = 0.0134) compared to Ciprofloxacin alone (LRV = 1.81, 98.44%, *p* = 0.2676). In contrast, adding 50 µg/mL to the antibiotic had no effect on CFU reduction (LRV_60 + 30_ = 1.94, 98.86%, *p* = 0.0689).

### 2.5. PVP–Curcumin Did Not Prevent Development of Antibiotic Resistance

**Figure 5:**
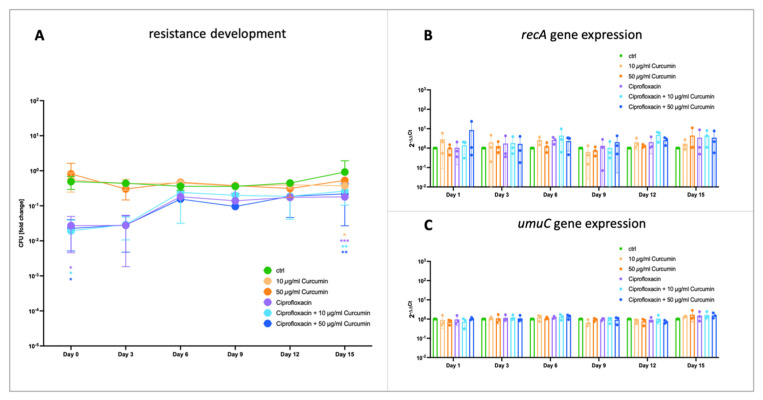
The impact of PVP–curcumin on Ciprofloxacin resistance development in *E. coli* K12. (**A**) The bacterial strain was treated every day with a sublethal dose of Ciprofloxacin and/or PVP–curcumin (either 10 or 50 µg/mL curcumin) and cultivated further in fresh medium. Every third day, CFUs were determined after treatment to assess bacterial growth and the efficacy of the antibiotic. While after six days, the antibacterial effect of Ciprofloxacin was reduced, there was no difference whether cells were pre-treated with PVP–curcumin or not. Values depicted are normalized to the starting bacterial concentration of 1 × 10^7^ CFU. (**B**) Expression of the bacterial SOS response initiator protein RecA—indicated as 2^−ΔΔCt^—was not significantly different in the overnight cultures of any of the treatment groups. (**C**) Likewise, *umuC* gene expression stayed consistent over the duration of 15 days in all conditions. Two-way ANOVA, Fisher’s least significant difference test, mean ± SD, *n* = 3, * *p* ≤ 0.05, ** *p* ≤ 0.01, *** *p* ≤ 0.001.

To assess the effect of PVP–curcumin on the development of antibiotic resistance, *E. coli* K12 was repeatedly exposed to a sublethal dose of Ciprofloxacin and/or two different doses of PVP–curcumin over the course of 15 days. On the day of the first treatment (day 0), CFU reduction was comparable in all groups that received Ciprofloxacin, regardless of pre-treatment with PVP–curcumin (Figure 5A; LRV_Ciprofloxacin_ = 1.39, 83.05%, *p* = 0.0349; LRV_Ciprofloxacin + 10 µg/mL Curcumin_ = 1.53, 85.79%, *p* = 0.0321; LRV_Ciprofloxacin + 50 µg/mL Curcumin_ = 1.42, 85.93%, *p* = 0.0333; significances refer to differences between control group and treatment groups on the respective day). Further, the susceptibility towards Ciprofloxacin was reduced similarly in all three groups over the timeline of the experiment (15 days). Mean LRVs and percent of reduction can be seen in Table 1. Additionally, the expression of the genes *recA* and *umuC* were investigated. However, due to the high number of cells necessary for RNA extraction, gene analysis was not performed directly after antibiotics/curcumin treatment but bacteria were allowed to grow overnight before being subjected to RNA extraction. Here, no differences among groups or timepoints were observed (Figure 5B,C). To summarize, PVP–curcumin did not prevent or delay the development of Ciprofloxacin resistance under the chosen test conditions. Even though the cells did aquire tolerance towards the antibiotic, this did not affect the expression of the investigated genes.

## 3. Discussion

Wound infections occur when pathogenic microorganisms invade the soft tissue in a wound or the surrounding tissue, causing inflammation and tissue damage. Often, small infections resolve on their own; however, severe ones require medical intervention, especially in aged patients or when co-morbidities such as type II diabetes are involved. Besides debriding the wound, the standard treatment for infected wounds is the topical (and, in some cases, systemic) administration of antibiotic drugs. Since resistances towards these drugs are steadily increasing, health care systems around the world are facing a major dilemma that requires the development of new and effective treatment approaches.

Due to its multi-target nature, aPDT has become widely recognized as a promising strategy to overcome this challenge. It was demonstrated numerous times that the bacterial burden of wounds could be efficiently reduced in pre-clinical animal models [4,17]. Furthermore, the risk of microbes developing resistance towards aPDT is considered to be low [18]. Over the years, various molecules have been identified as PSs, such as the phenolic compound curcumin, which has been used traditionally as herbal supplement, cosmetics ingredient, food flavoring and food coloring. Many positive properties have been attributed to it, such as being anti-inflammatory, immunomodulating and anti-cancerous, which earned it the reputation of being a highly versatile and promising compound [19,20]. However, recognition is rising that curcumin is a PAINS molecule (pan assay interference compound) that can interact with many biological assays and, thereby, distort results. Studies often fail to account for this fact and are lacking the appropriate controls. Therefore, literature should be critically reviewed and future research should take this into account [21].

Curcumin is considered safe for consumption, as clinical studies showed that ingesting 8 g per day for 3 months did not have negative side effects [22]. Still, its bioavailability is very low, with often undetectable plasma concentrations after oral administration [23]. Furthermore, it is lipophilic and, therefore, poorly water-soluble. It is often solubilized in solvents that could cause toxicity such as ethanol, methanol or dimethyl sulfoxide (DMSO) and it degrades rapidly [24]. Therefore, various strategies have been developed to improve its solubility, such as encasing curcumin in nanoparticles, liposomes, micelles or attaching it to other molecules. For example, loading Polylactide-co-glycolic acid (PLGA) or Polyethylene glycol (PEG) nanoparticles with curcumin significantly increased the bioavailability and half-time [25,26]. Here, we tested the water-soluble and highly stable compound PVP–curcumin. PVP is a synthetic polymer that is generally recognized as safe and widely used in cosmetics, food and pharmaceutics as thickening agent, film former, dispersing agent, etc. The first aim of the present work was to evaluate PVP–curcumin as PS for the potential application in aPDT for wound disinfection. We tested the effects in both Gram-negative (*E. coli* K12) and Gram-positive (*S. capitis*) strains and demonstrated, that both can be inactivated by the combination of blue light and PVP–curcumin. The degree of the antibacterial efficacy depended on the concentration of curcumin as well as on the wavelength and dose of blue light used. While *S. capitis* responded strongly even to small concentrations, *E. coli* K12 was only reduced by 1.09-log_10_ units when exposed to 10 µg/mL curcumin and 15 J/cm^2^ of 475 nm blue light (Figure 1A).

Changing the wavelength to 415 nm led to a stronger decrease of CFUs of 4.26-log_10_ units, even with a much lower light dose (2.2 J/cm^2^). Possible explanations are, on the one hand, that 415 nm lie closer to the absorption maximum of PVP–curcumin than 475 nm (the spectrum can be found in Supplemental Material Figure S1) and, on the other hand, that Gram-negative bacterial strains possess a different cell wall structure. They have an outer cell membrane that provides a stronger barrier for various molecules compared to Gram-positive strains [27]. Nonetheless, an uptake assay performed with *E. coli* K12 indicated that PVP–curcumin was taken up by these bacteria (Supplemental Material Figure S2).

In contrast to PVP–curcumin, unconjugated curcumin (dissolved in DMSO) has limited application potential due to its water-insolubility. Here, when attempting to perform aPDT with regular curcumin, concentrations higher than 10 µM (corresponding to 3.7 µg/mL) precipitated during experiments. No reduction in *E. coli* K12 was detected when irradiated with 15 J/cm^2^ of 475 nm blue light and 3.7 µg/mL regular curcumin (Supplemental Material Figure S5).

Additionally, we were interested in the use of 475 nm blue light as a light source for aPDT, as we and others demonstrated previously that 475 nm blue light enhanced wound healing and tissue regeneration. Proposed mechanisms include the release of nitric oxide, stimulation of growth factors, decrease of pro-inflammatory cytokines and increase of collagen [6,7,28,29,30].

Interestingly, aPDT exhibited a biphasic effect in both bacterial strains. The peak efficacy at 15 J/cm^2^ was seen at a curcumin concentration of 10 µg/mL in *E. coli* K12 and 50 µg/mL in *S. capitis*, whereas with 200 µg/mL, the efficacy was decreased in both strains. These data highlight the importance of investigating the dose–response relationship. In this case, the effect might be due to the antioxidant properties of curcumin surpassing and inactivating the ROS produced in the photoreaction.

Recently, curcumin has been suggested to possess inherent antimicrobial characteristics by disrupting the cell membrane and preventing FtsZ ring formation [31,32,33]. However, a large amount of published literature does not state where the curcumin was obtained from, the solvent used to dissolve it or, in the case of possible cytotoxic solvents, does not include solvent-only control groups. Hence, these results must be carefully interpreted. In the present work, we did not see any antibacterial effects of PVP–curcumin alone using concentrations up to 200 µg/mL (concentration refers to the absolute curcumin content) in *E. coli* K12 (Figure 1A).

Besides acting as a PS, there is some evidence that curcumin could influence the bacterial SOS response [10,11,12]. This pathway is activated when the initiator protein RecA recognizes DNA damage and, subsequently, facilitates the expression of genes involved in DNA repair by stimulating the autocatalytic cleavage of the transcriptional repressor LexA [14,34]. Depending on the amount of DNA binding sites for LexA, some genes are expressed early in the SOS response and others are reported to be translated in later stages [35]. For example, early genes include *recA* and *lexA* themselves, but also genes for high-fidelity DNA repair such as the nucleotide excision repair (NER) [13]. RecA itself is not only the initiator protein for this stress response, but also functions as a homologous recombinase. If the stress on the bacterial cell persists, lower fidelity DNA mechanisms are expressed, including the error-prone DNA polymerase V, which does not possess any proofreading activity and, thereby, contributes to mutagenicity [36,37]. Inhibiting the SOS response has been associated with preventing horizontal gene transfer of antibiotic resistance genes and virulence factors and increasing susceptibility towards antibiotics, even when resistance already occurred [37,38,39,40].

Ciprofloxacin is a fluoroquinolone antibiotic that inhibits the bacterial gyrase and DNA topoisomerase IV resulting in a stalled DNA replication and acts on Gram-negative as well as Gram-positive bacterial strains [41]. It has been shown numerous times to trigger the bacterial SOS response and was also observed in our gene expression analysis by increased *recA* expression (Figure 3) [38,42,43]. Blocking the SOS response makes bacteria more susceptible to DNA-damaging agents such as Ciprofloxacin and less likely to cope with environmental stresses.

Oda, in 1995, demonstrated that 7.8 µg/mL curcumin inhibited 50% of the UV-induced *umuC* expression in *E. coli* K12, the gene encoding a subunit of DNA polymerase V. Furthermore, mutagenesis was reduced by approximately 50% by 30 µg/mL curcumin [11]. There is also some evidence that curcumin can inhibit the ATPase activity of RecA in vitro [12]. Similarly, Bellio et al., in 2013, observed a decrease of a Levofloxacin-induced reporter gene under the control of the LexA repressor by 4 and 8 µg/mL curcumin. Planktonic *E. coli* suspensions were incubated with the antibiotic and curcumin for 60 min [10]. Matching the conditions of aPDT, we investigated the expression of the genes *recA* and *umuC* after 20 min of pre-treatment with PVP–curcumin (10 and 50 µg/mL curcumin concentrations) and a subsequent 10 min Ciprofloxacin treatment (Figure 3). The increase of *recA*’s expression caused by the sublethal dose of Ciprofloxacin was dampened by pre-incubating *E. coli* K12 with PVP–curcumin. However, we observed high intraexperimental variations of the Ciprofloxacin-induced *recA* expression ranging from 4.23- to 430-fold. Still, when looking at individual experimental runs, there was a noticeable trend towards a decline in expression caused by the PVP–curcumin treatment. When comparing those experimental groups to the Ciprofloxacin-only group instead of the untreated control, the expression was decreased to 0.73- and 0.41-fold, respectively (see Supplemental Material Figure S3). Interestingly, we also observed a reduced *recA* expression with the mono-treatment of 50 µg/mL curcumin but not with 10 µg/mL. There were no distinct changes in *umuC*’s expression, most likely due to the short duration of the treatment.

We additionally analyzed the gene expression after aPDT; however, using 15 J/cm^2^ of 475 nm blue light did not cause an increase in *recA* or *umuC*’s expression; therefore, no effect of curcumin could also be observed in the aPDT groups (Supplemental Material Figure S4). In contrast to our findings, Rapacka-Zdonczyk et al. 2019 did observe an initiation of the SOS response in *S. aureus* by blue light irradiation, but they used a much higher light dose (150 J/cm^2^) and a lower wavelength of blue light (411 nm), which presumably generated more ROS than in the present study [44].

Furthermore, we investigated whether PVP–curcumin could potentiate the antibacterial effects of a sublethal Ciprofloxacin dose in *E. coli* K12 (Figure 4). When we first pre-treated bacterial cells with PVP–curcumin before 10 min of antibiotic exposure, CFU reduction was minimally enhanced from 1.05-log_10_ units (only Ciprofloxacin) to 1.46-log_10_ (10 µg/mL curcumin + Ciprofloxacin) and 1.42-log_10_ (50 µg/mL curcumin + Ciprofloxacin). Increasing the incubation durations to 60 min pre-treatment and 30 min antibiotic therapy resulted in a slightly potentiated efficacy from 1.81-log_10_ (only Ciprofloxacin) to 2.4-log_10_ units (10 µg/mL curcumin + Ciprofloxacin). On the contrary, 50 µg/mL did not show a similar result (LRV = 1.94). Overall, there was a minor trend visible; however, the effect might not translate into meaningful clinical benefits. Further research with different incubation protocols might provide more insight to explore the full potential of this approach.

During any type of antibacterial treatment for in vivo wound disinfection—including aPDT and antibiotic drugs—it is likely that only a fraction of bacteria will be exposed to a sublethal dose and survive. Still, DNA damage might occur and activate the SOS response, which has been associated with mutagenesis through the induction of error-prone DNA repair pathways. This can contribute to the emergence of antibiotic resistances [45]. We tested the potential of PVP–curcumin to prevent the development of resistance by exposing *E. coli* K12 to a low dose of Ciprofloxacin with or without PVP–curcumin pre-treatment for 15 consecutive days (Figure 5). Bacterial cells showed tolerance towards Ciprofloxacin after 6 days. There were no differences in the PVP–curcumin-treated groups, demonstrating that RecA inhibition had no effect in this experimental setting. Analysis via qPCR revealed no differences in *recA* or *umuC* expression. Since in this study, RNA was harvested after overnight incubation to reach sufficient cell numbers (and, therefore, RNA amount); it is suggested that the genes involved in the SOS response were already downregulated to a physiological level at the time of RNA isolation. This is also indicated by the experimental group that was solely treated with Ciprofloxacin and did not show elevated *recA* expression.

To sum up, the present study aimed to investigate several aspects of the antibacterial treatment of the novel compound PVP–curcumin, namely aPDT and modulation of the bacterial SOS response. This combination would be of high interest for the treatment of wound infections due to the growing challenge of antibiotic resistance. PVP–curcumin is a water-soluble compound that acts as a PS when irradiated with blue light. There is some evidence that it can influence the initiator protein of the SOS response; however, we could not see a substantial impact in more clinically relevant settings. Further research of this complex and multifaceted pathway is needed and crucial for developing strategies to reverse antibiotic resistance or prevent its emergence in the future.

## 4. Materials and Methods

### 4.1. Bacterial Strains and PVP–Curcumin

PVP–curcumin was provided by PLANTA Naturstoffe Vertriebs GmbH, Vienna, Austria. Weight proportion of curcumin (Carl Roth GmbH + Co., KG, Karlsruhe, Germany) ranged between 1 and 3.2%. Curcumin complex bond to PVP (Kollidon^®^ 17 PF, BASF Pharma, Ludwigshafen, Germany) ensured the monomolecular distribution and the retaining of the properties of curcumin. Indicated curcumin concentrations (e.g., 10 or 50 µg/mL) refer to the curcumin part of the compound PVP–curcumin. The compound was dissolved in double-distilled water.

Two bacterial strains were used in the present study: Gram-negative *Escherichia coli* K12 (Addgene, Watertown, MA, USA) and Gram-positive *Staphylococcus capitis*, kindly provided by Dr. Kristjan Plätzer from the Paris Lodron University Salzburg, Austria.

### 4.2. Cultivation and Quantification of Bacteria

Bacterial strains were cultivated in lysogeny broth (LB) medium (VWR International, Radnor, PA, USA) and grown at 37 °C under aerobic conditions and 200 rpm in a shaking incubator (Edmund Bühler TH15, Hechingen, Germany). Before starting an experiment, planktonic cultures of the strains were diluted in fresh medium to initiate exponential growth, incubated for 30–40 min and pelletized (3 min, 13,000× *g*, MiniSpin^®^, Eppendorf, Hamburg, Germany) for resuspension in Dulbecco’s Phosphate Buffered Saline (PBS, without MgCl_2_ and CaCl_2_, Sigma-Aldrich, St. Louis, MI, USA). Next, bacterial cell concentration was estimated via optical density measurements at 600 nm (OD_600_) using the relations *E. coli* K12: OD_600_ of 1.0 = 4 × 10^8^ CFU/mL and *S. capitis*: OD_600_ of 1.0 = 1.6 × 10^7^ CFU/mL. Cell count was adjusted to the needed concentration depending on the experiment. CFUs were quantified following antibiotic treatment or photodynamic therapy by serially diluting the samples 1:10 in PBS and transferring several 20 µL droplets onto LB–agar plates (AppliChem GmbH, Darmstadt, Germany). After overnight incubation at 37 °C, colonies were counted and compared to an untreated control group. Decreased CFU numbers were calculated out of six technical replicates per experimental group and expressed as mean log-reduction value (LRV) using Equation (1):

(1) $$LRV\;\left[-\right]={log}_{10}\frac{{CFU}_{ctrl}\;\left[\frac{CFU}{mL}\right]}{{CFU}_{treatment}\;\left[\frac{CFU}{mL}\right]}$$

### 4.3. Antibacterial Photodynamic Therapy

A total of 250 µL of bacterial suspension containing 1 × 10^7^ CFU/mL was incubated for 20 min with PVP–curcumin (curcumin concentration: 10 or 50 µg/mL) in 24-well plates at 37 °C, 100 rpm. Next, the LED device (REPULS Lichtmedizintechnik GmbH, Vienna, Austria) was placed on the lid. The devices emitted blue light with either 475 ± 13 nm (measured half-width at half-maximum) with an intensity of 50 mW/cm^2^ or 415 ± 11 nm with 7.3 mW/cm^2^. They were characterized with a USB2000 spectrometer (Ocean Optics, Orlando, FL, USA) and a GL Spectis 1.0 device (Just Normlicht GmbH, Weilheim, Germany), respectively. The 475 nm LED device radiates a pulsed light with a pulse rate of 50% and a pulse repetition frequency of 2.5 Hz, whereas the 415 nm LED device emits continuous light. Duration of irradiation was chosen to result in a fluency of 7.5, 15, 30 and 45 J/cm^2^ for 475 nm, whereas 415 nm blue light was administered at a dose of 2.2 J/cm^2^. After the treatment, planktonic suspensions were handled as described above.

### 4.4. Potentiation of Ciprofloxacin

A planktonic *E. coli* K12 suspension with 1 × 10^7^ CFU/mL was prepared in several microcentrifuge tubes as described above. In the respective groups, 10 or 50 µg/mL curcumin was added before incubating the samples at 37 °C, 150 rpm for 20 or 60 min. Next, 0.4 µg/mL of Ciprofloxacin (Sigma-Aldrich, St. Louis, MI, USA) was added and an additional incubation period of either 10 or 30 min followed. Control groups received an equal volume of PBS. Antibiotic efficacy was assessed by applying the droplet method previously explained. Further, to determine gene expression after sublethal Ciprofloxacin exposure, the experiment was repeated using higher cell numbers to yield enough mRNA. A total of 1 × 10^8^ CFUs were pre-treated with 10 or 50 µg/mL curcumin for 20 min and, subsequently, with 0.4 µg/mL Ciprofloxacin for 10 min. Four samples per experimental group were prepared and pooled after the treatment to obtain 4 × 10^8^ CFUs in total. These were then pelletized by centrifuging for 3 min at 13,000× *g* and resuspended in TRI Reagent^®^ (MRC, Cincinnati, OH, USA) before freezing the samples at −80 °C until further processing.

### 4.5. Gene Expression Analysis by RT-qPCR

The RNA of bacterial samples was isolated by using the phenol–chloroform extraction method according to a standard protocol. Briefly, bacterial cells were disrupted and homogenized in TriReagent^®^ (Molecular Research Center, Inc., Cincinnati, OH, USA), RNA was extracted with chloroform, then precipitated with isopropanol and the pelleted RNA was washed with ethanol. After drying, it was resuspended in a suitable volume of nuclease-free water and the quantity measured using Nanodrop One^©^ (ThermoFisher Scientific, Waltham, MA, USA). Equal amounts where then treated with DNase I (RQ1 RNase-Free DNase kit, Promega, Madison, WI, USA) before performing cDNA Synthesis using the OneScript^®^ Plus cDNA synthesis kit (Applied Biological Materials Inc., Richmond, Canada). Details of the primers applied in RT-qPCR can be found in Table 2. Primers were analyzed for efficiency beforehand. The gene *yccT* served as the housekeeping gene. The amplification of DNA was monitored via intercalation of Blue S’Green dye (Biozym Scientific GmbH, Hessisch Oldendorf, Germany) and detected by qTOWER^3^ G (Analytik Jena GmbH, Jena, Germany). The following temperature protocol was used: 3 min at 95 °C, 3 s at 95 °C, 30 s at annealing temperature. The last two steps were repeated for 39 cycles and, lastly, a melting curve with 0.5 °C increments was performed while the signal was obtained at each temperature step.

### 4.6. Antibiotic Resistance Development

**Figure 6:**
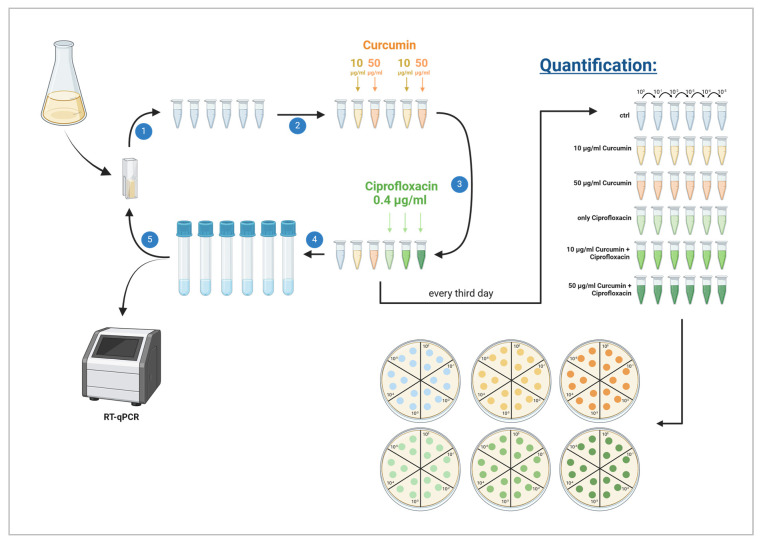
Experimental setup to test the influence of PVP–curcumin on antibiotic resistance development. (1) On day 0, the optical density of a planktonic overnight culture of *E. coli* K12 was determined and six microcentrifuge tubes were prepared, each containing the desired cell concentration. (2) PVP–curcumin was added to the respective tubes to reach a curcumin concentration of 10 or 50 µg/mL. Bacterial cells were incubated for 20 min at 37 °C. (3) Three tubes were treated with 0.4 µg/mL Ciprofloxacin for 10 min. Additionally, on day 0 as well as every third day, each experimental group was serially diluted 1:10 for the quantification of bacterial cell numbers after the treatments. Next, three drops of each dilution were transferred to agar plates and incubated at 37 °C overnight. (4) Immediately after antibiotic treatment, vented tubes filled with 1 mL culture medium were inoculated with a 10 µL aliquot of the respective experimental group. Every third day, the rest of the overnight cultures were harvested for qPCR analysis. (5) At the beginning of each day, the optical density of all individual tubes was measured to assess the cell number. Image created with Biorender.com.

To evaluate a potential effect on the development of antibiotic resistance, *E. coli* K12 cultures were exposed daily, first to PVP–curcumin, and then to a sublethal concentration of Ciprofloxacin over the course of 15 days. On the first day (day 0), the cell count of a planktonic culture was determined via OD_600_ as described above. Six microcentrifuge tubes were filled with 1 mL containing 1 × 10^7^ CFU in LB medium and the respective groups received 10 or 50 µg/mL curcumin. After 20 min of incubation at 37 °C with 100 rpm, 0.4 µg/mL Ciprofloxacin was added for an additional 10 min. Next, 10 µL of the treated bacterial suspensions were transferred into individual vented snap tubes filled with 1 mL fresh LB medium for overnight incubation. On the next day, OD_600_ was determined for each experimental group and six microcentrifuge tubes containing 1 × 10^7^ CFU were prepared again. Then, the same antibiotic and/or PVP–curcumin treatment was repeated. Control groups without the compound and/or without the antibiotic were included. Every third day, the bacterial cell count was identified by serially diluting aliquots for plating as described above. Additionally, overnight cultures were harvested for RNA extraction and RT-qPCR. A graphical scheme of the experimental setting can be seen in Figure 6.

### 4.7. Statistics

Statistical significance was tested using the Kruskal–Wallis test with Dunn’s multiple comparison test or two-way ANOVA with Fisher’s least significant difference test by using GraphPad Prism 10 (GraphPad Software, Inc., Boston, MA, USA), as indicated in the respective figure descriptions. Significance was accepted at *p* ≤ 0.05. Outliers were identified via ROUT method with the value of Q set to 0.5%.

## 5. Conclusions

Wound infections pose a significant healthcare challenge due to increasing antibiotic resistance. aPDT is a promising alternative since it targets a broad spectrum of microbes. We aimed to test the photosensitizing abilities of the stable, water-soluble compound PVP–curcumin as an alternative to regular curcumin. We demonstrated effectiveness against both Gram-negative as well as Gram-positive bacterial strains with low PVP–curcumin concentrations and a relatively low dose of blue light (15 J/cm^2^). Curcumin is also reported to prevent the initiation of the bacterial SOS response by interacting with the protein RecA or hindering its expression. Blocking this DNA repair pathway should decrease the mutagenicity of bacteria and make them more susceptible to bactericidal treatments. However, we did not observe clinically relevant effects in the tested translational assays. While the modulation of the SOS response requires further research, PVP–curcumin is a promising candidate for aPDT as a PS.

## Figures and Tables

**Table 1:** Mean log reduction values (LRV) and percent of reduction (%) of *E. coli* K12 treated with the respective reagents.

	Ctrl		10 µg/mL Curcumin		50 µg/mL Curcumin		Ciprofloxacin		Ciprofloxacin + 10 µg/mL Curcumin		Ciprofloxacin + 50 µg/mL Curcumin	
	LRV	%	LRV	%	LRV	%	LRV	%	LRV	%	LRV	%
Day 0	0.00	0.00	−0.04	−21.75	−0.10	−18.36	1.39	83.05	1.53	85.79	1.42	85.93
Day 3	0.00	0.00	0.03	0.06	0.21	26.42	1.55	93.13	1.26	92.85	1.46	93.00
Day 6	0.00	0.00	−0.09	−28.95	−0.11	−36.89	1.18	59.63	0.73	30.14	1.06	64.71
Day 9	0.00	0.00	−0.01	−2.83	−0.02	−5.43	0.92	63.90	0.59	45.15	0.92	75.06
Day 12	0.00	0.00	0.26	33.27	0.26	39.18	1.12	89.13	0.80	68.45	0.70	64.86
Day 15	0.00	0.00	0.22	22.27	0.07	−6.90	0.95	87.03	0.45	50.77	0.78	69.84

**Table 2:** Sequences, concentrations and annealing temperatures of primers used for RT-qPCR.

Gene	Primer Sequence	Primer Concentration	Annealing Temperature
*yccT* (housekeeper)	sense: 5’-TTA ATA CCC AGC TCA TCA ACC A-3’ antisense: 5’-CTG TCA TCT GCC ATC ATC GT-3’	400 nM	63 °C
*recA*	sense: 5’-GCT GAA ATT CTA CGC CTC TG-3’ antisense: 5’-CAG TCG CAT TCG CTT TAC C-3’	400 nM	61 °C
*umuC*	sense: 5’-TTA TCT GTT CCC GCT CGT-3’ antisense: 5’-ATA TCC CTG CTG TCC TGA G-3’	400 nM	63 °C

## Data Availability

The data presented in this study are available on request from the corresponding author.

## Supplementary Materials

The following supporting information can be downloaded at https://www.mdpi.com/article/10.3390/ijms25116140/s1.
