# Supplementary material for: The Multifaceted Actions of PVP–Curcumin for Treating Infections
Source: Int J Mol Sci. 2024 Jun 2;25(11):6140. doi: 10.3390/ijms25116140 (PMC11172534; doi:10.3390/ijms25116140)
Supplement: Supplementary file 1 [file ijms-25-06140-s001.zip › supplemental material/supplemental material_curcumin paper_first revision.pdf]

Supplemental Material

1. Absorption spectrum of PVP-Curcumin

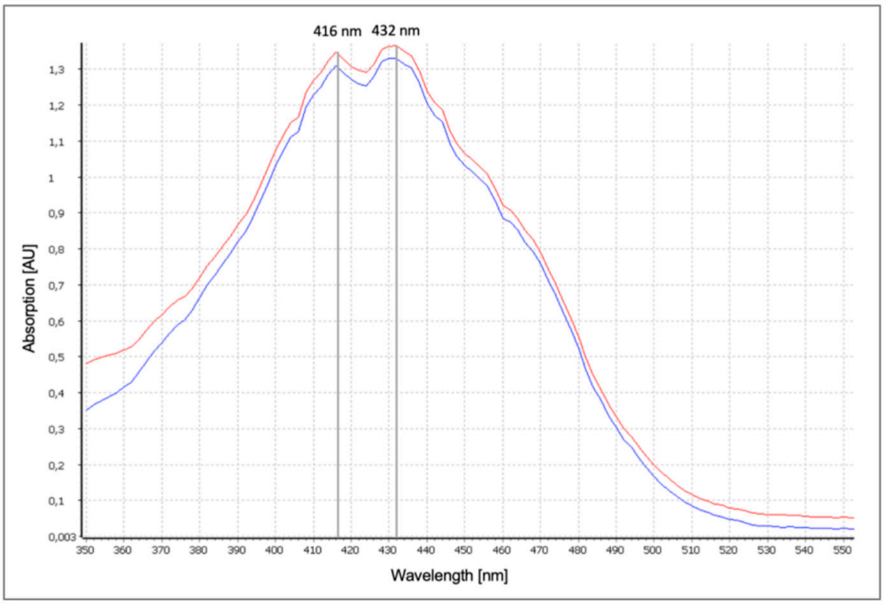

***Figure S1. Absorption spectrum of PVP-Curcumin dissolved in ddH$_2$O**. The absorption spectrum of PVP-Curcumin (Curcumin concentration: 5 µg/ml) was measured in a plate reader (POLARstar® Omega, BMG Labtech, Ortenberg, Germany) using a wavelength range from 350-550 nm. Two absorption maxima were recorded: 416 and 432 nm. pH value changes did not influence the peaks (data not shown). The resulting extinction coefficient of both wavelengths is 95,800 l · mol^{-1} · cm^{-1}. y-axis shows absorption in arbitrary units (AU) and x-axis shows wavelength in nm. The red line represents raw data and the blue one shows blank (ddH$_2$O) corrected values.*

2. PVP-Curcumin was taken up by *E. coli* K12 after 20 min incubation

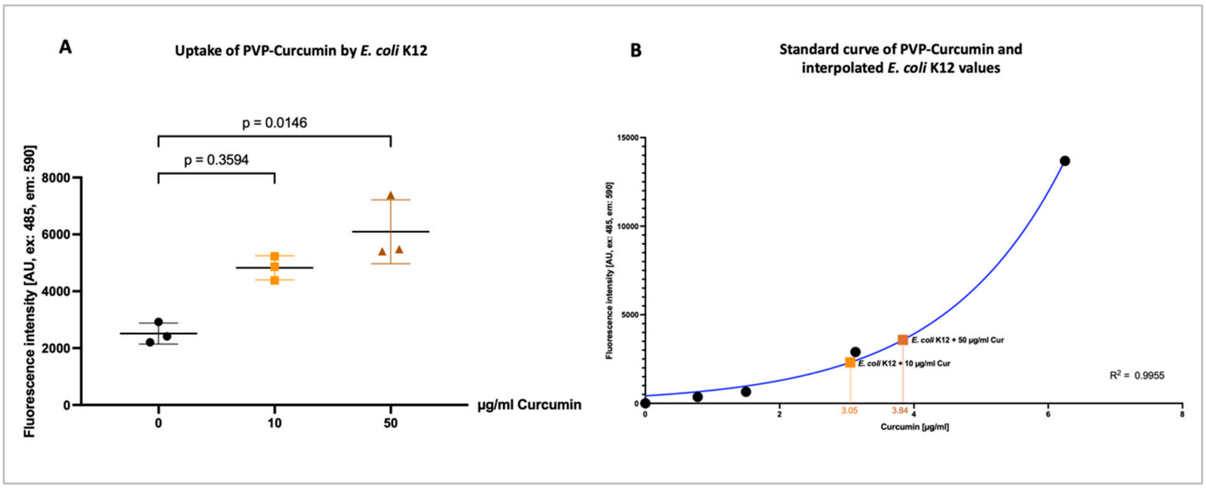

***Figure S2: Investigating if E. coli K12 can take up PVP-Curcumin of the surrounding environment. (A)** Planktonic cultures in the exponential growth phase were pelletized via centrifugation (MiniSpin®, Eppendorf SE, Hamburg, Germany) at 13 000 rpm for 3 min. Growth-medium was discarded and cells were resuspended in PBS. Next, they were incubated with PVP-Curcumin (10 or 50 µg/ml referring to the Curcumin proportion of the compound) for 20 min at 37°C in a shaking incubator. The control group received ddH$_2$O instead. Then, three washing steps were conducted by pelletizing and resuspending in PBS again. The washed cells were then transferred to a black 96-well plate and fluorescence was measured on the plate reader (POLARstar® Omega, BMG Labtech, Ortenberg, Germany) using 485 nm excitation and 590 nm emission wavelengths. Bacterial cells without PVP-Curcumin pre-incubation had a mean fluorescence intensity of 2514 arbitrary units (AU) which was increased to 4825 and 6092 AU after pre-treatment with 10 or 50 µg/ml Curcumin respectively. This indicates that PVP-Curcumin was taken up by the Gram-negative bacterial strain E. coli K12 or that it at least accumulated at the cell*

wall. It is important to note that the experiment was repeated without using bacterial cells and no fluorescence was detected in the PVP-Curcumin groups, verifying that the compound did not precipitate and form a pellet of itself (data not shown). ***(B)*** *The obtained fluorescence values from the uptake assay were interpolated into a standard curve measured of multiple PVP-Curcumin concentrations, which presented as an exponential growth curve. Here, 10 µg/ml Curcumin corresponded to 3.05 µg/ml and 50 µg/ml Curcumin to 3.84 µg/ml. The assay was repeated three times with two technical replicates. Mean ± SD. Kruskal-Wallis test, Dunn's multiple comparison test. Statistical significance was accepted at* $p \leq 0.05$.

3. Ciprofloxacin induced expression of the gene *recA* was reduced with PVP-Curcumin compared to Ciprofloxacin alone in *E. coli* K12

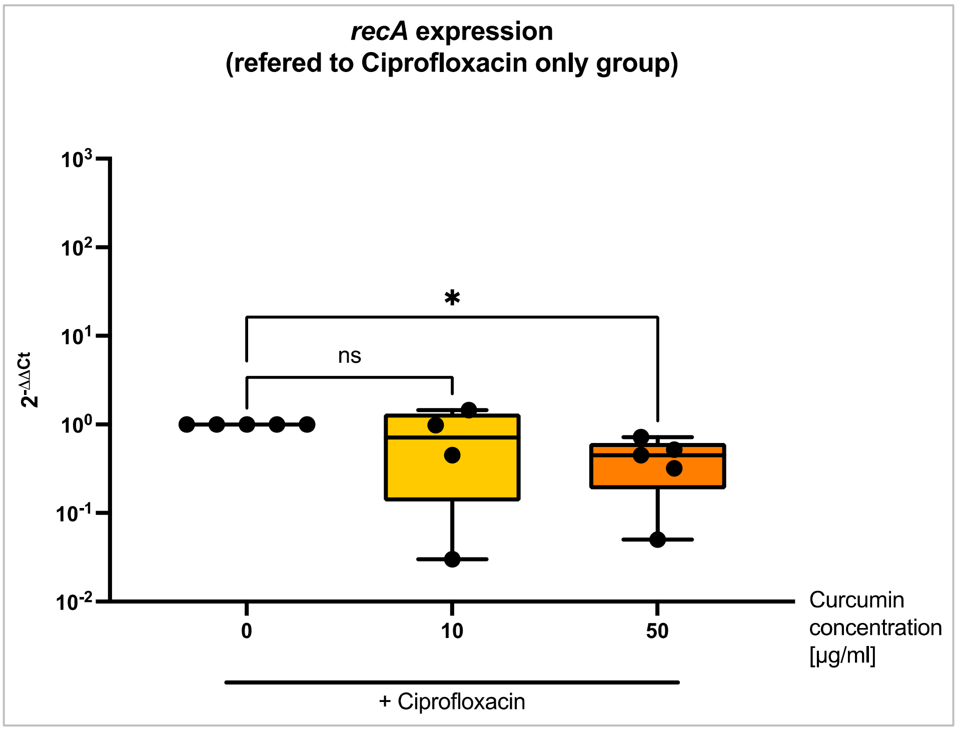

Figure S3. Expression the gene recA which encodes the initiator protein of the bacterial SOS response RecA was decreased by PVP-Curcumin. *In contrast to comparing the gene expression levels to an untreated control group, here, Ciprofloxacin treatment served as reference and was set to 1. Treatment with 10 µg/ml Curcumin reduced values to 0.73 and 50 µg/ml to 0.41. Housekeeping gene: yccT. Box and whisker blots indicating median, minimum and maximum values, Kruskal-Wallis test, Dunn's multiple comparison test, ROUT method outlier test, n= 5,* $* \ p \leq 0.05$

4. 475 nm blue light irradiation did not induce expression of the genes *recA* and *umuC*

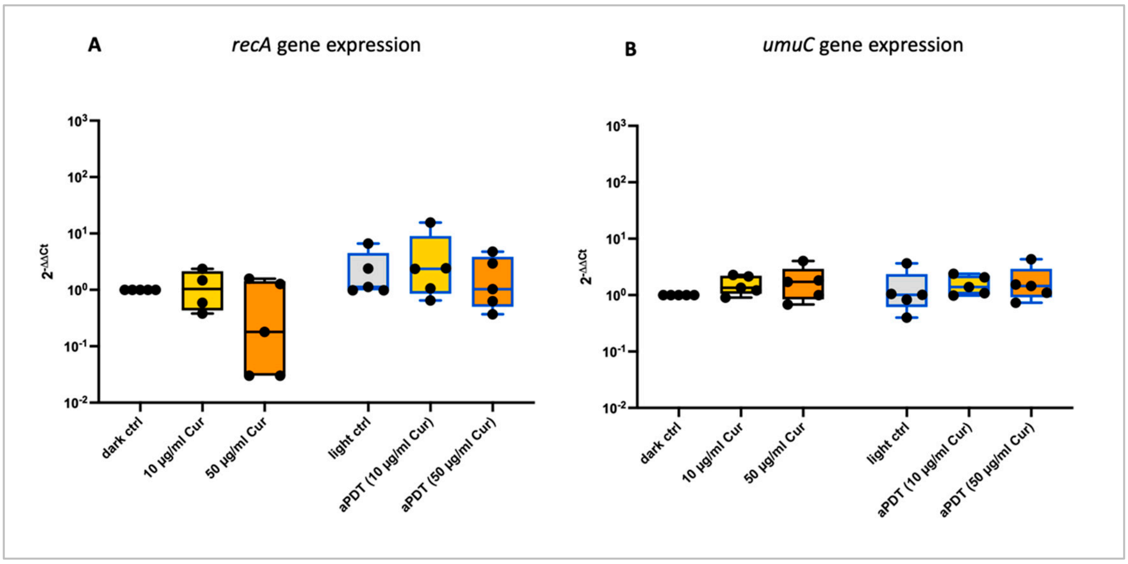

***Figure S4. Gene expression of recA and umuC after antibacterial Photodynamic therapy (aPDT).** (**A**) 15 J/cm² of 475 nm blue light irradiation (indicated as light ctrl) did not increase gene expression of recA, therefore there were no noticeable differences after aPDT using 10 or 50 µg/ml Curcumin. (**B**) Similarly, expression of umuC remained comparable to non-treated cells (termed dark ctrl). Housekeeping gene: yccT. Box and whisker blots indicating median, minimum and maximum values, Kruskal-Wallis test, Dunn's multiple comparison test, ROUT method outlier test, n= 5*

5. aPDT using unconjugated Curcumin did not result in bacterial inactivation

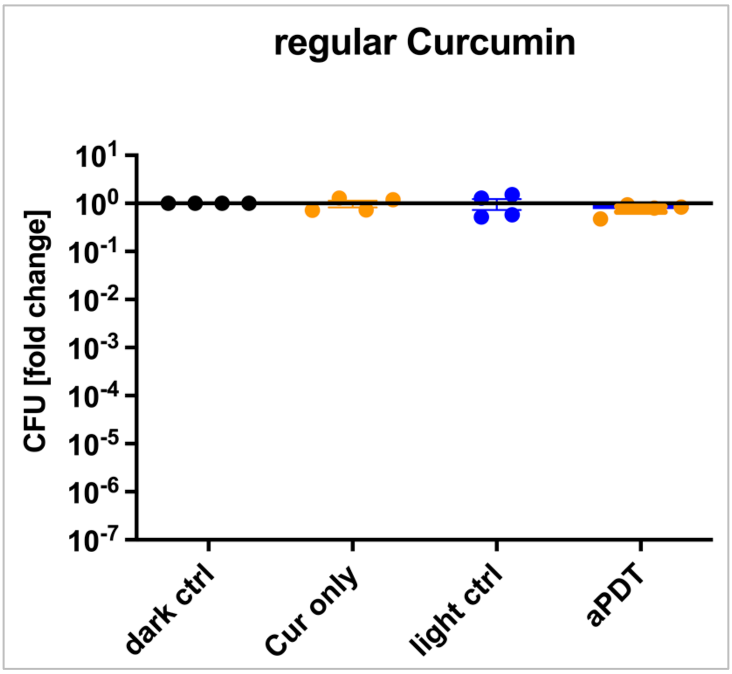

***Figure S5: Colony forming units (CFU) of E. coli K12 were not reduced by aPDT using unconjugated Curcumin and 475 nm blue light.** Cells were brought into the exponential growth phase and then pre-incubated with 10 µM (corresponds to 3.7 µg/ml) regular Curcumin (dissolved in DMSO) for 60 min before they were exposed to 15 J/cm² blue light. No effect was observed under the tested conditions. Mean ± SEM, n = 4*

6. Light intensity measurement of LED devices

For light intensity measurements, the light devices were placed 2 cm above the sensor head, which resembles the height of a standard cell culture plate, in such a way that the light emitting diode was directly above the sensor area and the resulting intensity was recorded. The devices emitted blue light with either 475 ± 13 nm (measured half width at half maximum) with an intensity of 50 mW/cm^2 or 415 ± 11 nm with 7.3 mW/cm^2. They were characterized with a USB2000 spectrometer (Ocean Optics, Orlando, United States) and a GL Spectis 1.0 device (Just Normlicht GmbH, Weilheim, Germany), respectively.
