# Supplementary figures and images for: The Multifaceted Actions of PVP–Curcumin for Treating Infections
Source: Int J Mol Sci. 2024 Jun 2;25(11):6140. doi: 10.3390/ijms25116140 (PMC11172534; doi:10.3390/ijms25116140)

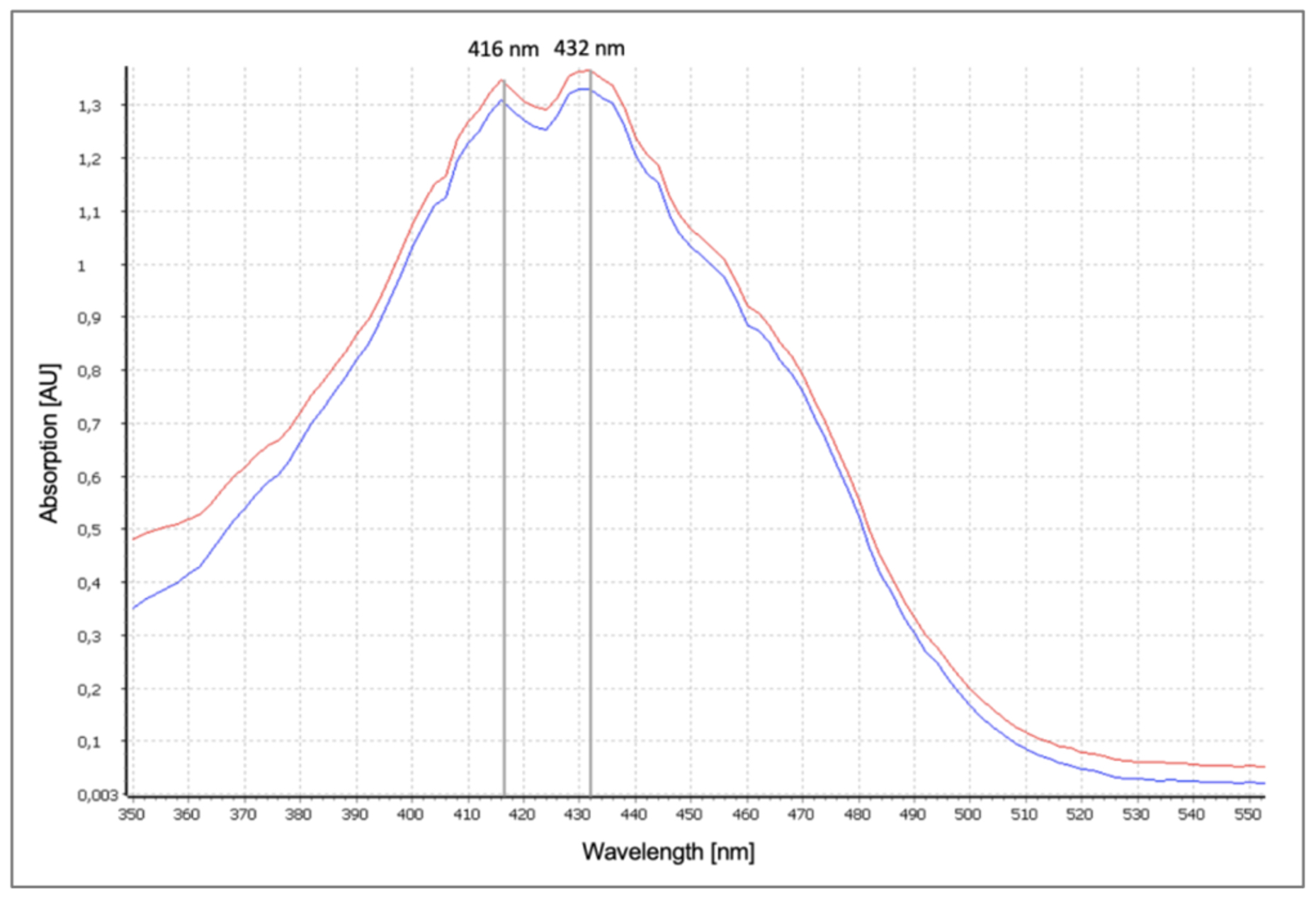

Supplement: Supplementary file 1 [file ijms-25-06140-s001.zip › supplemental material/jpeg figures/figure S1_Absorption spectrum PVP Curcumin.jpg]

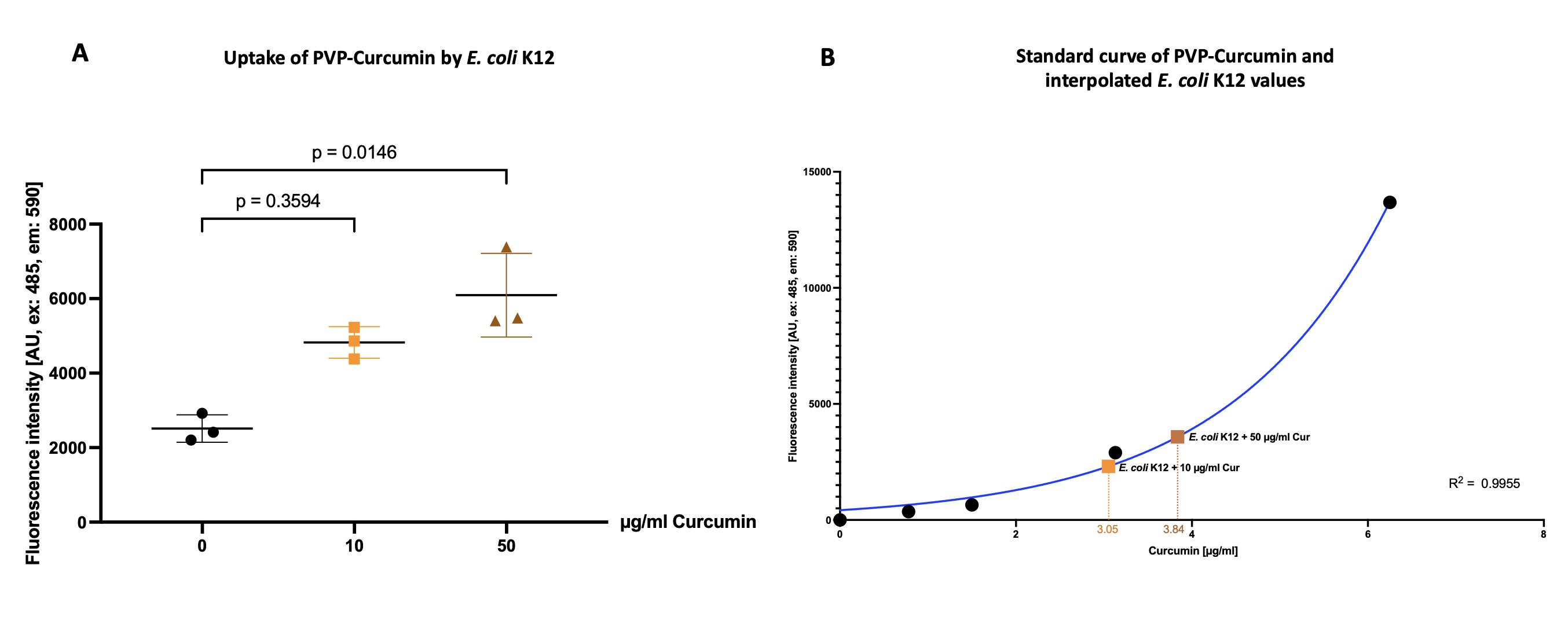

Supplement: Supplementary file 1 [file ijms-25-06140-s001.zip › supplemental material/jpeg figures/figure S2_PVP Curcumin Uptake.jpg]

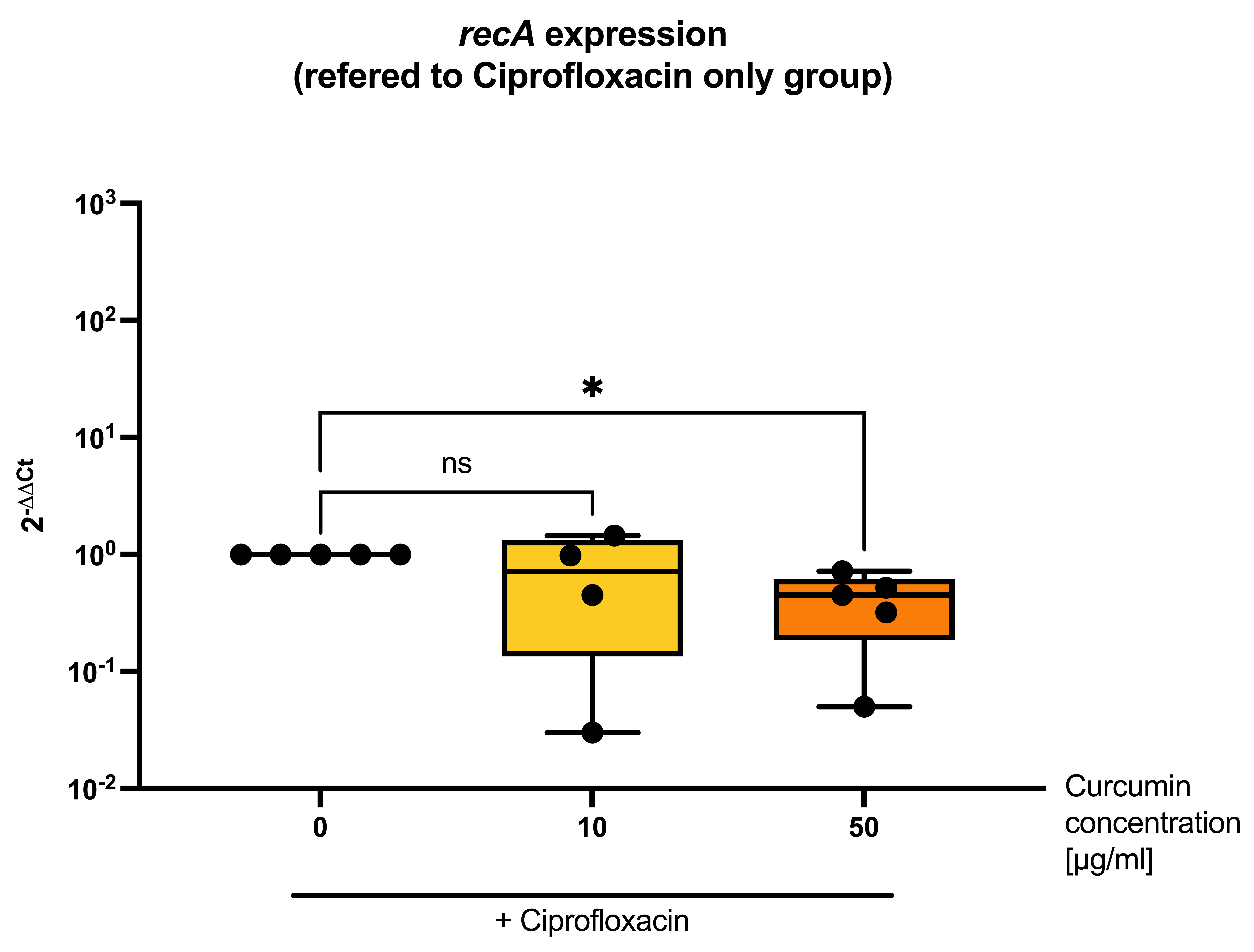

Supplement: Supplementary file 1 [file ijms-25-06140-s001.zip › supplemental material/jpeg figures/figure S3_Gene expression normalized to cipro only group.jpg]

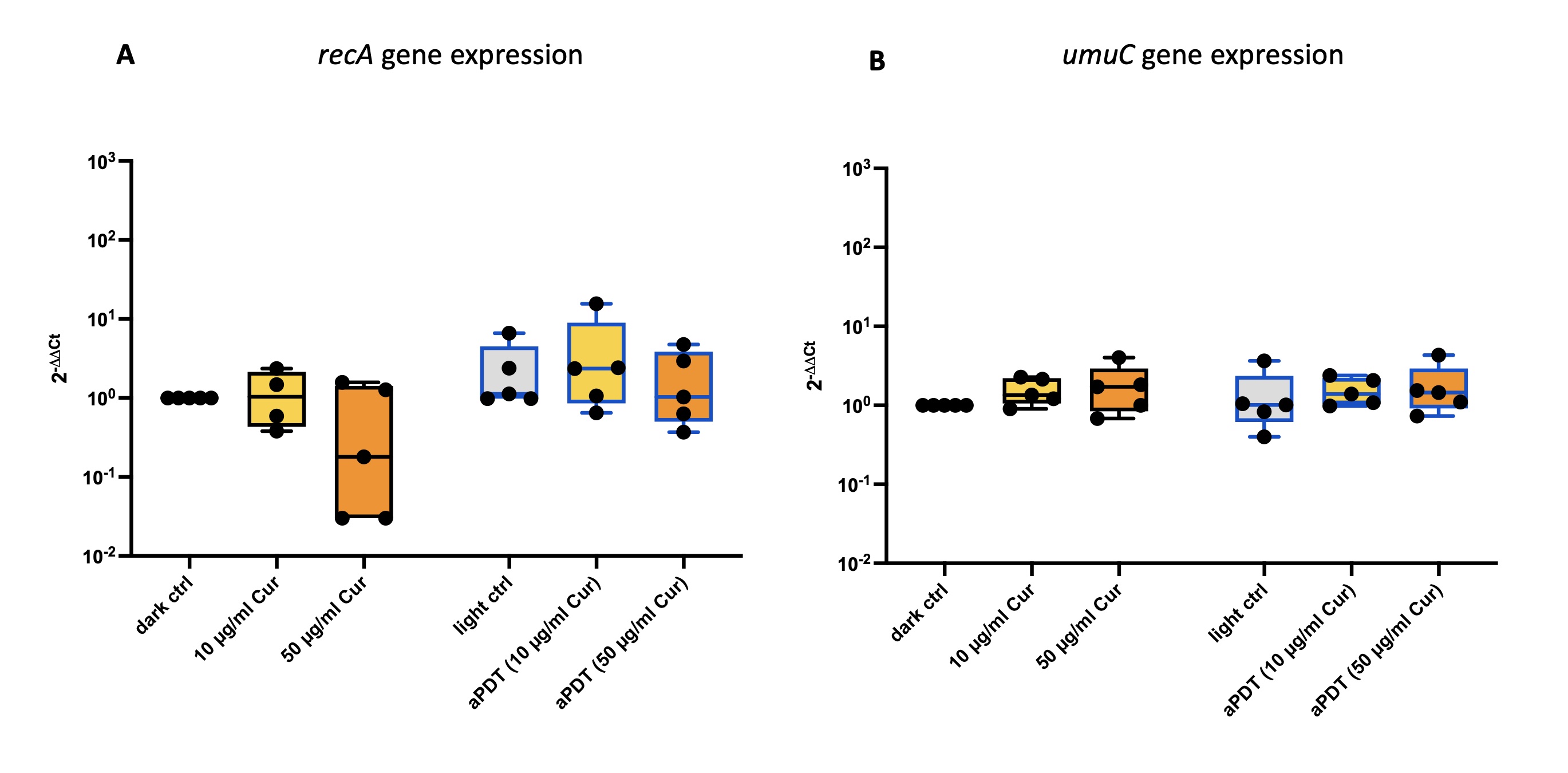

Supplement: Supplementary file 1 [file ijms-25-06140-s001.zip › supplemental material/jpeg figures/figure S4_Gene expression after aPDT.jpg]

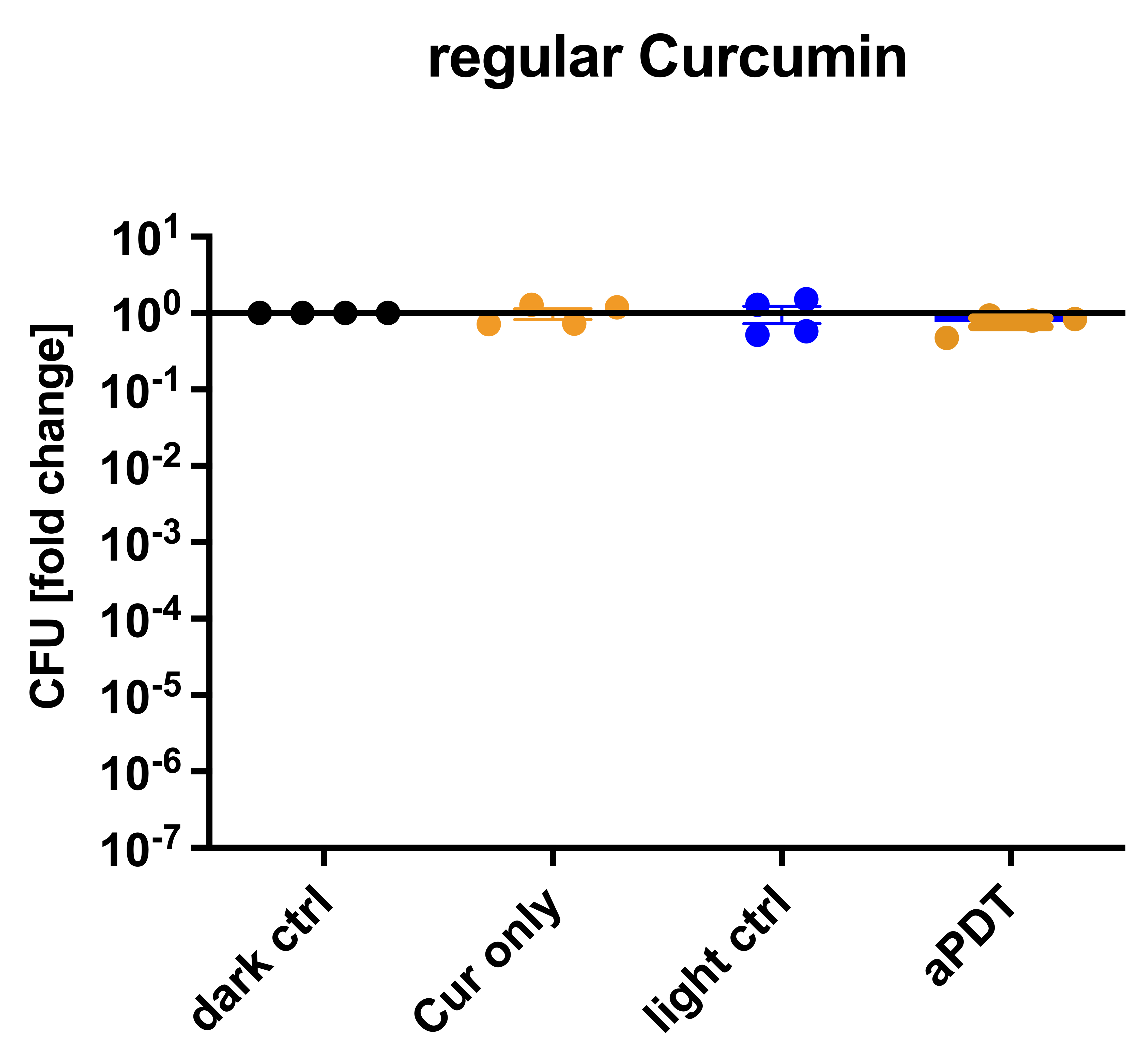

Supplement: Supplementary file 1 [file ijms-25-06140-s001.zip › supplemental material/jpeg figures/figure S5_aPDT regular Curcumin.jpg]

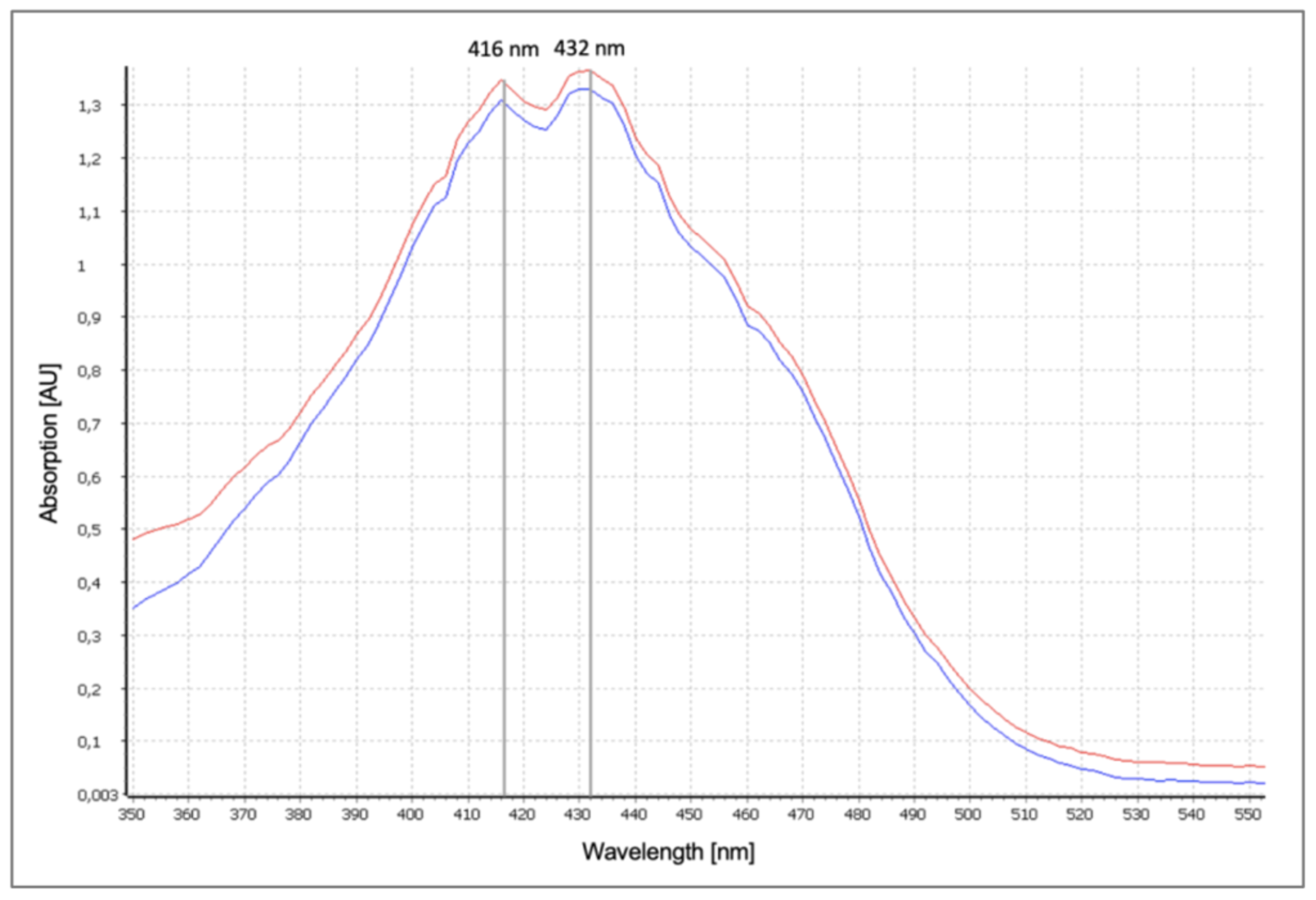

Supplement: Supplementary file 1 [file ijms-25-06140-s001.zip › supplemental material/png figures/figure S1_Absorption spectrum PVP Curcumin.png]

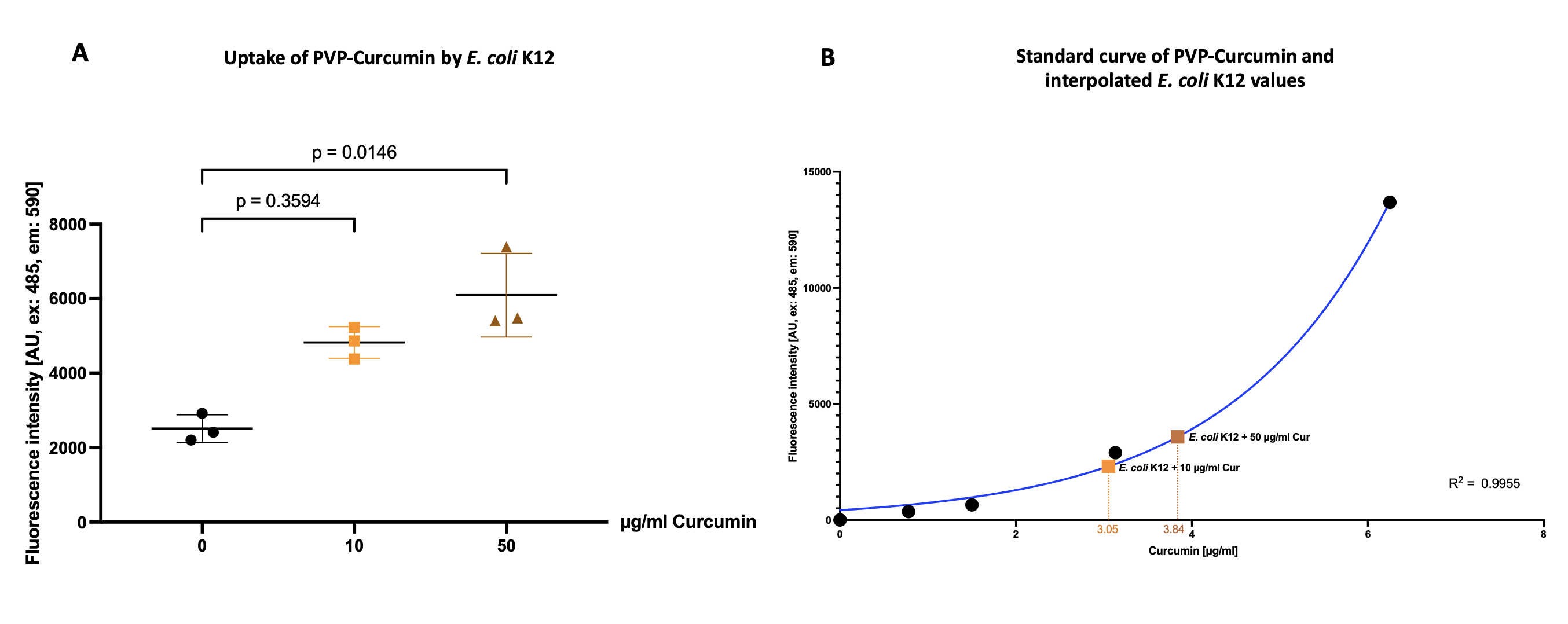

Supplement: Supplementary file 1 [file ijms-25-06140-s001.zip › supplemental material/png figures/figure S2_PVP Curcumin Uptake.png]

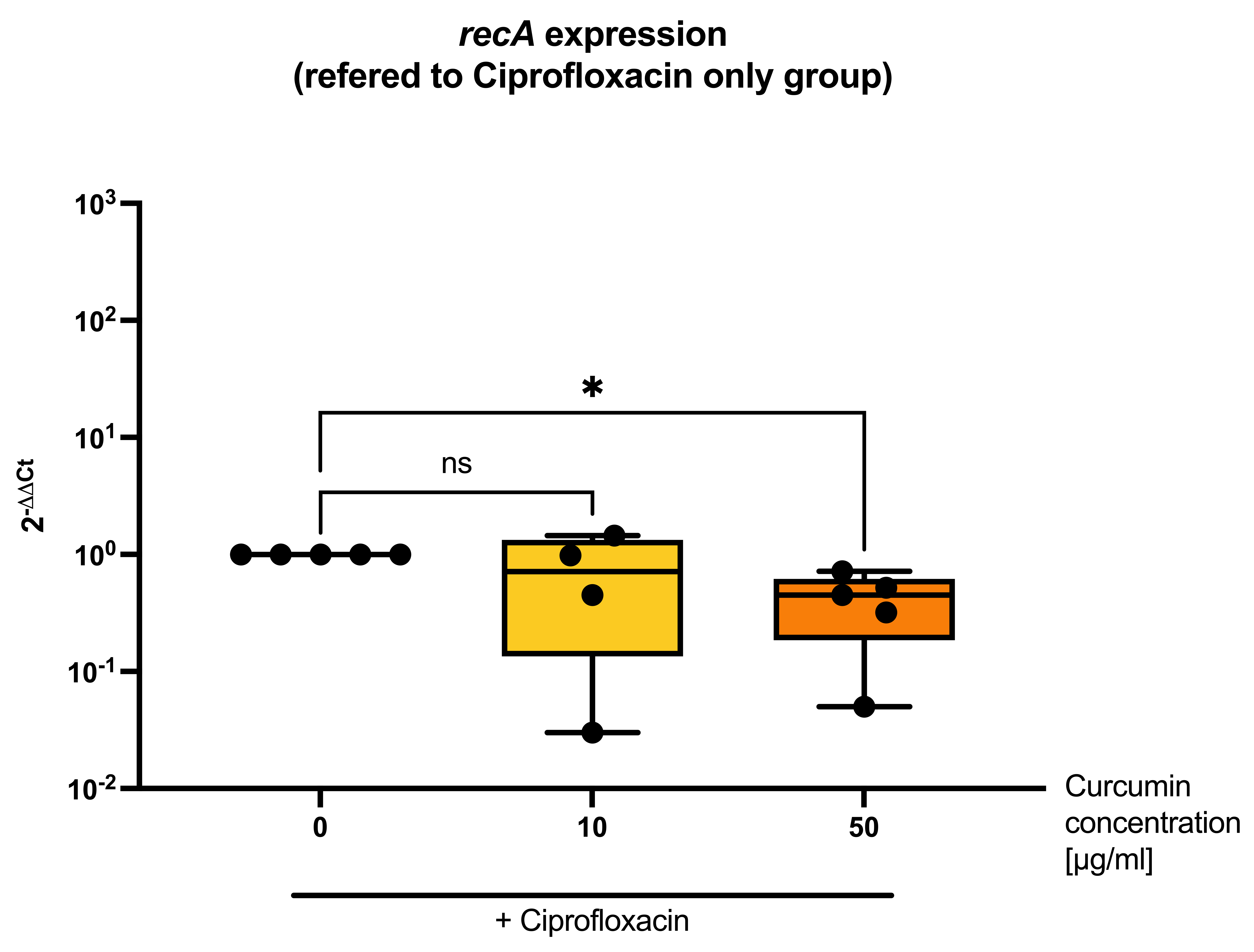

Supplement: Supplementary file 1 [file ijms-25-06140-s001.zip › supplemental material/png figures/figure S3_Gene expression normalized to cipro only group.png]

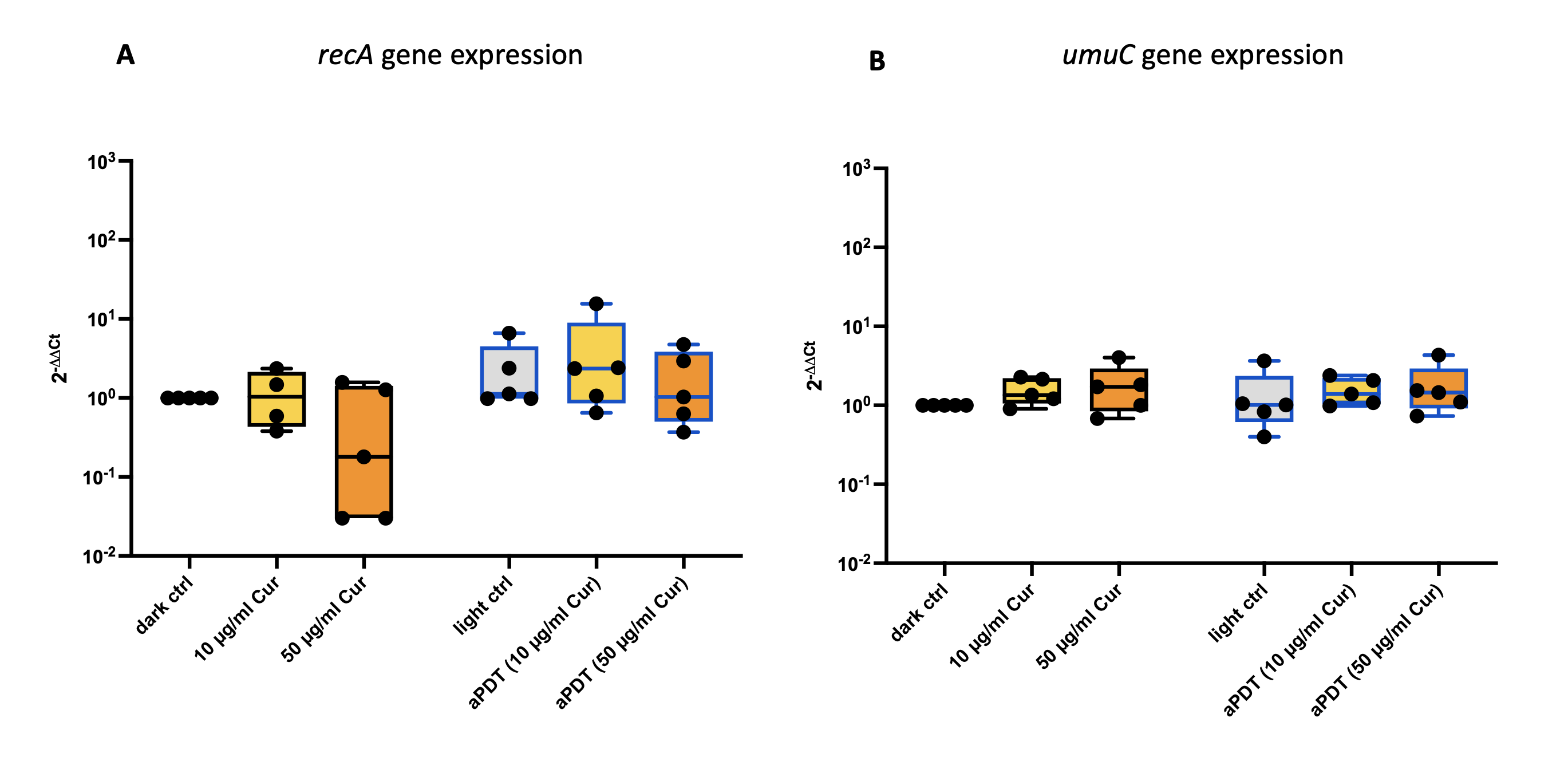

Supplement: Supplementary file 1 [file ijms-25-06140-s001.zip › supplemental material/png figures/figure S4_Gene expression after aPDT.png]

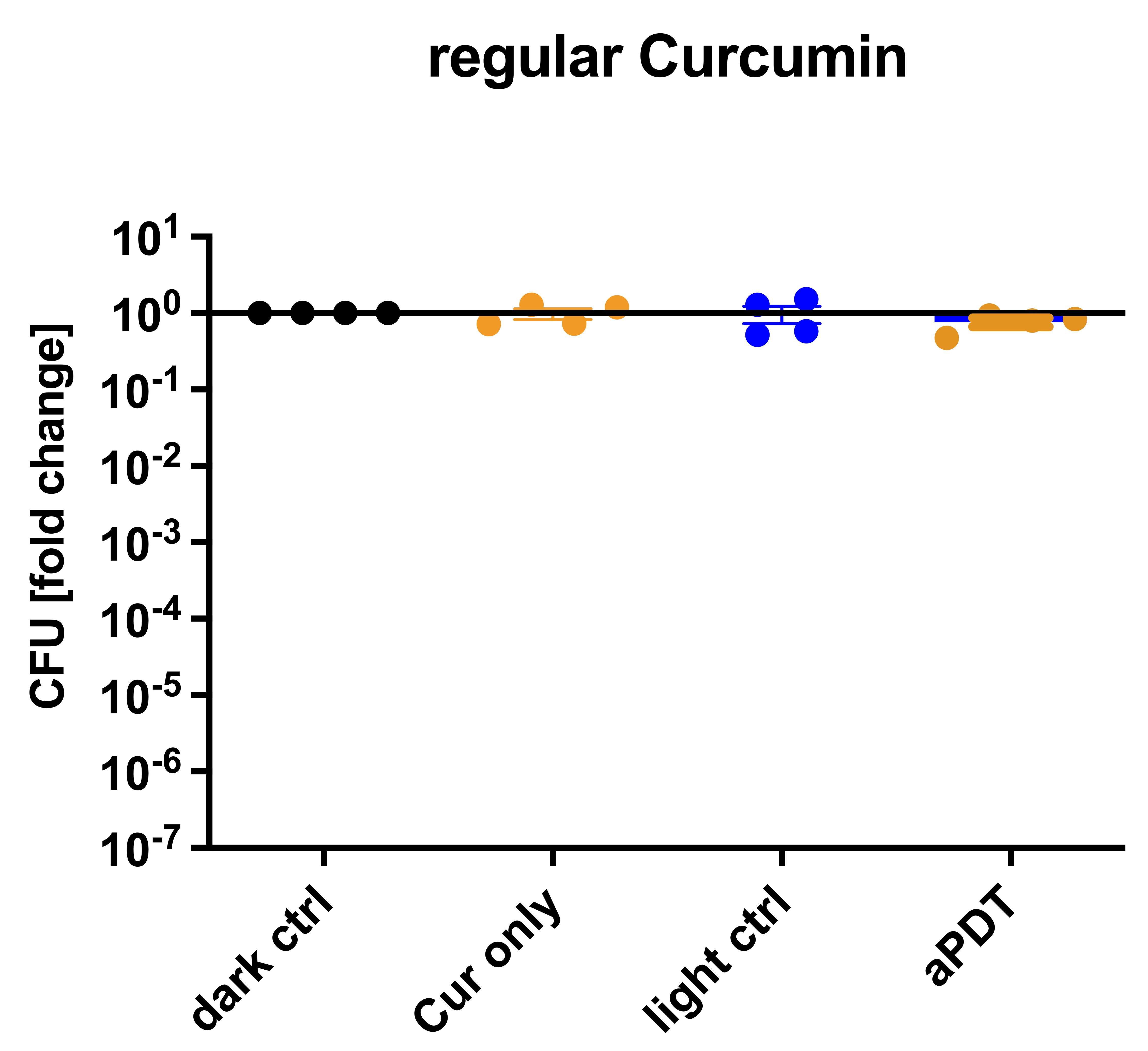

Supplement: Supplementary file 1 [file ijms-25-06140-s001.zip › supplemental material/png figures/figure S5_aPDT regular Curcumin.png]
